# Head-to-head comparison of [^18^F]CHL2205 *versus* [^18^F]T008 as PET radioligands to study brain cholesterol homeostasis in rodent models and nonhuman primates

**DOI:** 10.1007/s00259-026-07937-9

**Published:** 2026-05-30

**Authors:** Sébastien Goutal, Fabien Caille, Françoise Piguet, Maud Goislard, Sophie Amargier-Barrial, Sarah Leterrier, Solène Marie, Michel Bottlaender, Nicolas Tournier

**Affiliations:** 1 Laboratoire d’Imagerie Biomédicale Multimodale (BioMaps), Université Paris-Saclay, CEA, CNRS, Inserm, Service Hospitalier Frédéric Joliot, Orsay, 91401 France; 2 Innovation Unit GENOV, Paris Brain Institute, Inserm U 1127, CNRS UMR 7225, Sorbonne Université, Paris, 75013 France

**Keywords:** Alzheimer’s disease, Cholesterol, CYP46A1, PET imaging, [¹⁸F]CHL-2205, [¹⁸F]T008, Lipid metabolism, Neuroimaging

## Abstract

**Purpose:**

Cholesterol 24-hydroxylase (C24H) is central to brain cholesterol metabolism and is thought to be altered in many neurodegenerative diseases, including Alzheimer’s disease (AD). Only two fluorinated C24H-targeting PET radioligands, [¹⁸F]CHL-2205 and [¹⁸F]T008, have recently entered clinical use. Direct comparison is needed to guide radiotracer selection for future translational studies.

**Methods:**

Dynamic PET imaging was performed using either [¹⁸F]CHL-2205 or [¹⁸F]T008 in parallel conditions in cynomolgus macaques, healthy rats, the TgF344-AD rat model, and C24H-overexpressing mice. Arterial blood sampling enabled full kinetic modeling in healthy rats and nonhuman primates. An image-derived input function was validated in rats, and the specific binding was evaluated under C24H saturation using soticlestat.

**Results:**

[¹⁸F]CHL-2205 and [¹⁸F]T008 showed similar metabolism profiles in either macaques or rats. In macaques, brain uptake values (*V*_T_, Logan plot analysis) were 1.6-fold higher for [¹⁸F]CHL-2205 relative to [¹⁸F]T008. Healthy rats showed a similar pattern, with [¹⁸F]CHL-2205 exhibiting 1.5-fold higher brain *V*_T_. In rats, soticlestat blockade revealed C24H-specific binding for both ligands, with an estimated binding potential (BPND) 1.3-fold higher for [¹⁸F]CHL-2205 compared to [¹⁸F]T008. In TgF344-AD rats, brain uptake increased by 1.9-fold with [¹⁸F]CHL-2205 versus 1.6-fold with [¹⁸F]T008 compared to wild-type controls. In C24H-overexpressing mice, [¹⁸F]CHL-2205 detected measurable increases in brain signal in several brain regions (up to 1.5-fold), while [¹⁸F]T008 did not in the same animals.

**Conclusion:**

Across species and models, both radioligands demonstrated appropriate characteristics for imaging C24H in vivo. However, [¹⁸F]CHL-2205 generally displayed higher specific binding and appeared more sensitive to detect moderate increases in C24H expression.

**Supplementary Information:**

The online version contains supplementary material available at https://doi.org/10.1007/s00259-026-07937-9.

## Introduction

Cholesterol homeostasis in the brain is crucial for normal neuronal integrity and function. Dysregulation of cholesterol metabolism has been reported in several neurological conditions, including epilepsy, stroke, and neurodegenerative diseases such as Alzheimer’s disease (AD) and Parkinson’s disease (PD) [1].

In the brain, cholesterol 24-hydroxylase (cytochrome P450 46A1, CYP46A1, C24H) is responsible for catabolizing cholesterol into 24-hydroxycholesterol, which is further transported out of the brain across the blood–brain barrier (BBB) [2]. Therefore, C24H function is a key determinant of the elimination of cholesterol from the brain and represents an appealing biomarker of brain cholesterol metabolism. Recently, the availability of C24H-targeting PET radioligands has enabled the study of the pathophysiologic role of brain cholesterol metabolism in several neurological conditions [3].

**Fig. 1:**
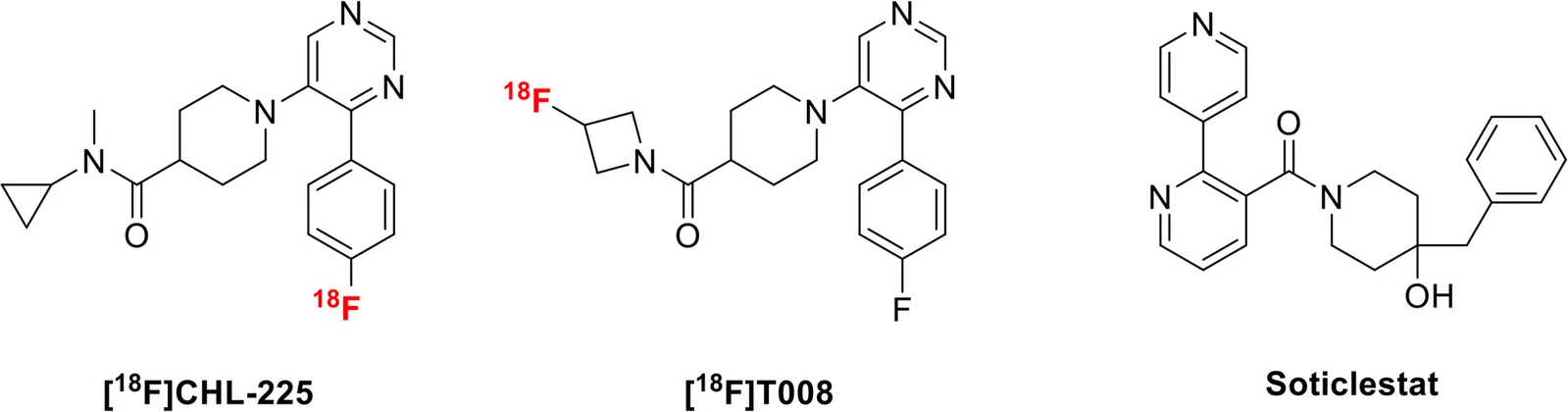
Chemical structures of [^18^F]CHL-2205, [^18^F]T008 and soticlestat

The PET radioligands [^18^F]CHL-2205 vs. [^18^F]T008 (Fig. 1) are the only compounds to have reached clinical status and have been validated as radiopharmaceuticals for imaging C24H in humans [4, 5]. [^18^F]CHL-2205 vs. [^18^F]T008 first underwent state-of-the-art preclinical validation in healthy rats, in C24H-deficient mice, or using a pharmacological blocking/occupancy study with the reference C24H ligand soticlestat (TAK-935, Fig. 1) in nonhuman primates [4, 6, 7]. Moreover, both radiotracers are fluorinated and represent appealing candidates to design large-scale clinical PET imaging protocols.

However, the selection of one or the other radiopharmaceutical for future translational research on brain cholesterol homeostasis should be based on a rational basis. Notably, the respective sensitivities of [^18^F]CHL-2205 and [^18^F]T008 for detecting changes in C24H expression in vivo have not been compared under parallel conditions. Moreover, abnormal cholesterol homeostasis in the brain often results in increased C24H density, as observed in AD and PD [3, 8].

The present study aimed to compare the PET kinetics and specific binding of [^18^F]CHL-2205 with those of [^18^F]T008 in healthy non-human primates and rats. Then, the increase in the PET signal observed in models of C24H overexpression was compared across two rodent models to test the sensitivity of both radiotracers under parallel conditions.

**Fig. 2:**
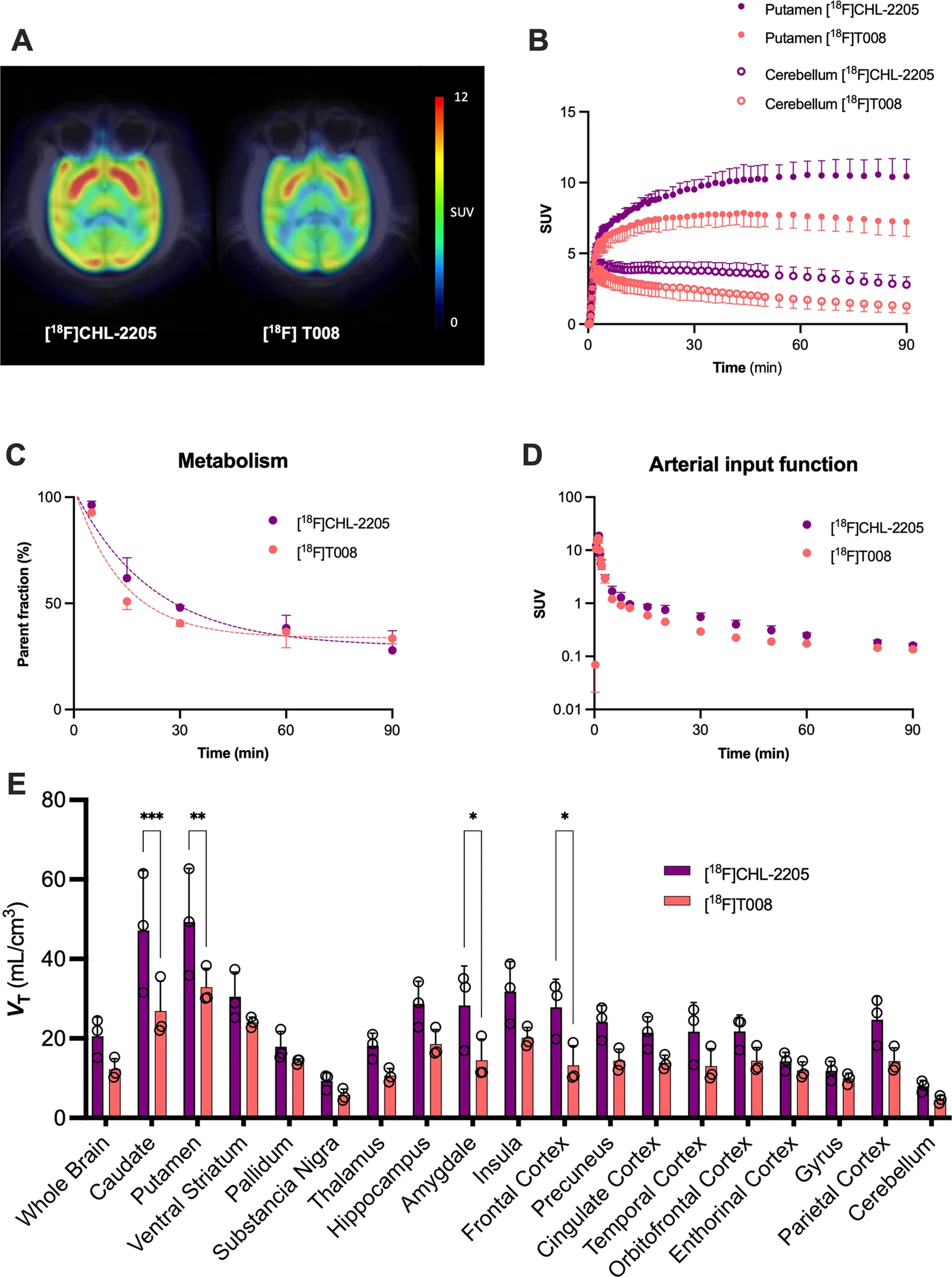
Comparison of [^18^F]CHL-2205 versus [^18^F]T008 in nonhuman primates. Representative brain PET images (summed 0-90 min) are displayed in **A**. Mean time-activity curves obtained in the putamen and cerebellum are shown in **B** (*n* = 3 per radiotracer). The mean metabolism profiles are shown in **C** and mean metabolite-corrected arterial input function are shown in **D**. Corresponding brain volume of distribution (*V*_T_, mean±S.D), estimated in selected brain regions using the Logan graphical method are reported in **E**. Significant differences are shown as *p < 0.05, **p < 0.01, and ***p < 0.001

## Materials and methods

### Chemistry, radiochemistry

The chemical structure of the tested ligands is shown in Fig. 1. The radiosynthesis of [^18^F]CHL-2205 ([^18^F]*N*-cyclopropyl-1-(4-(4-fluorophenyl)pyrimidin-5-yl)-*N*-methylpiperidine-4-carboxamide, *aka* [^18^F]cholestify) and [^18^F]T008 (3-[^18^F]fluoroazetidin-1-yl){1-[4-(4-fluorophenyl)pyrimidin-5-yl]piperidin-4-yl}methanone) were performed according to literature procedures on a Trasis All-in-One module [4, 6]. Soticlestat was obtained from Sigma-Aldrich (France).

**Fig. 3:**
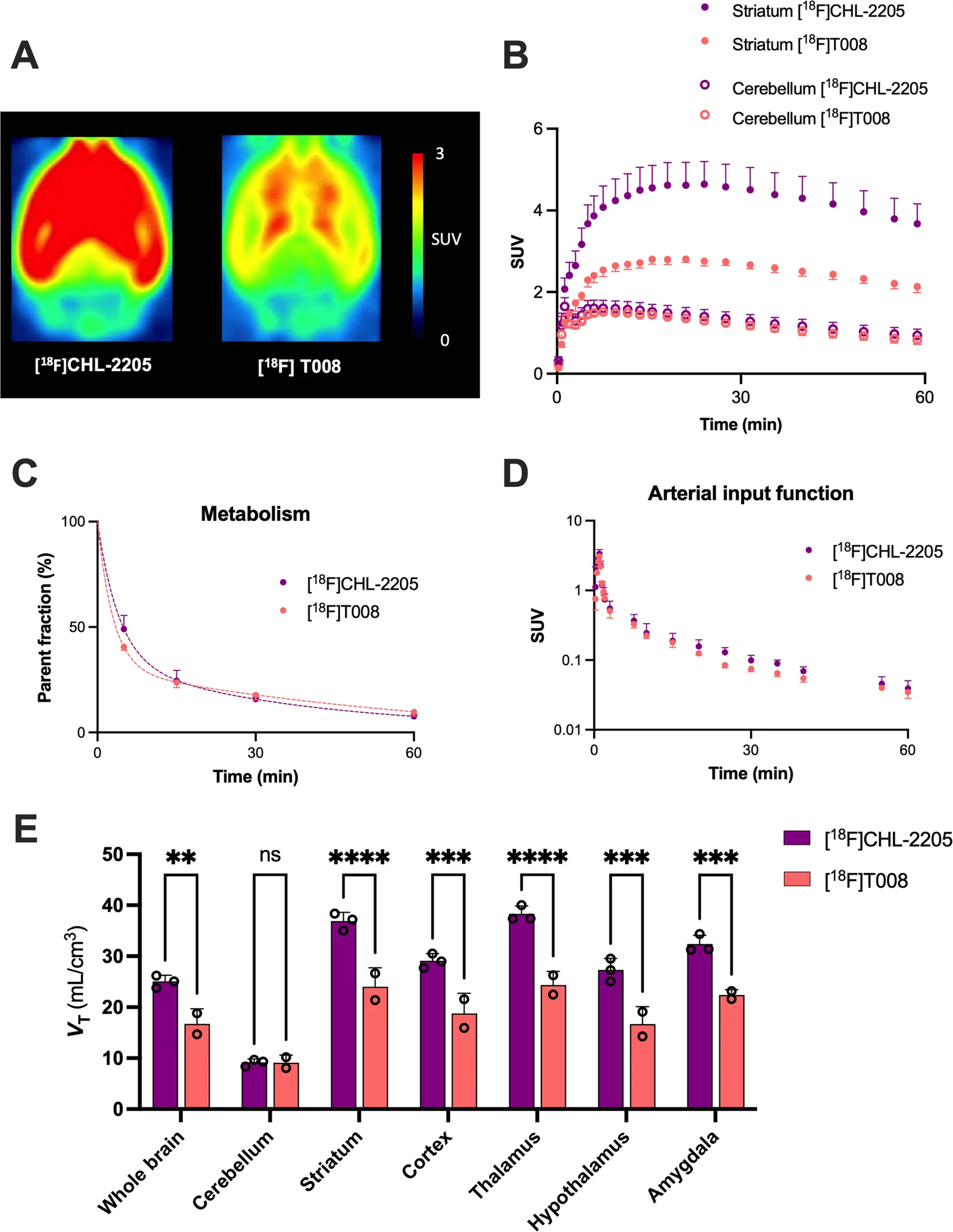
Comparison of [^18^F]CHL-2205 vs [^18^F]T008 in control rats. Representative brain PET images (summed 0-60 min) are displayed in **A**. Mean time-activity curves obtained in the putamen and cerebellum are shown in **B**. The mean metabolism profiles are shown in **C** and the mean metabolite-corrected arterial input functions are shown in **D**. Corresponding brain volume of distribution (*V*_T_), estimated in selected brain regions using the Logan graphical method are reported in **E**. Data obtained for [^18^F]CHL-2205 (*n* = 3) or [^18^F]T008 (*n* = 2) are reported as mean ± S.D. Significant differences are shown as ***p < 0.001 and ****p < 0.0001

### Animal housing and ethics

Animals were housed under standard experimental conditions: room temperature (20 ± 2 °C), a 12-hour light/12-hour dark cycle, provided with water and food *ad libitum*. They were kept in social groups with environmental enrichment. A 7-day acclimatization period was observed prior to any experiments. All procedures complied with European directives on the protection and use of laboratory animals (Council Directive 2010/63/EU, French Decree 2013–118). The experimental protocol was evaluated and approved by a local ethics committee for animal use and sanctioned by the French government (approval numbers APAFIS #44238-2023072111212158 v2 for rodents and APAFIS #41733-2023031614569134 v1 for non-human primates). The animal experiment data reported in this study comply with the ARRIVE 2.0 (Animal Research: Reporting in Vivo Experiments) guidelines. No randomization was performed.

**Fig. 4:**
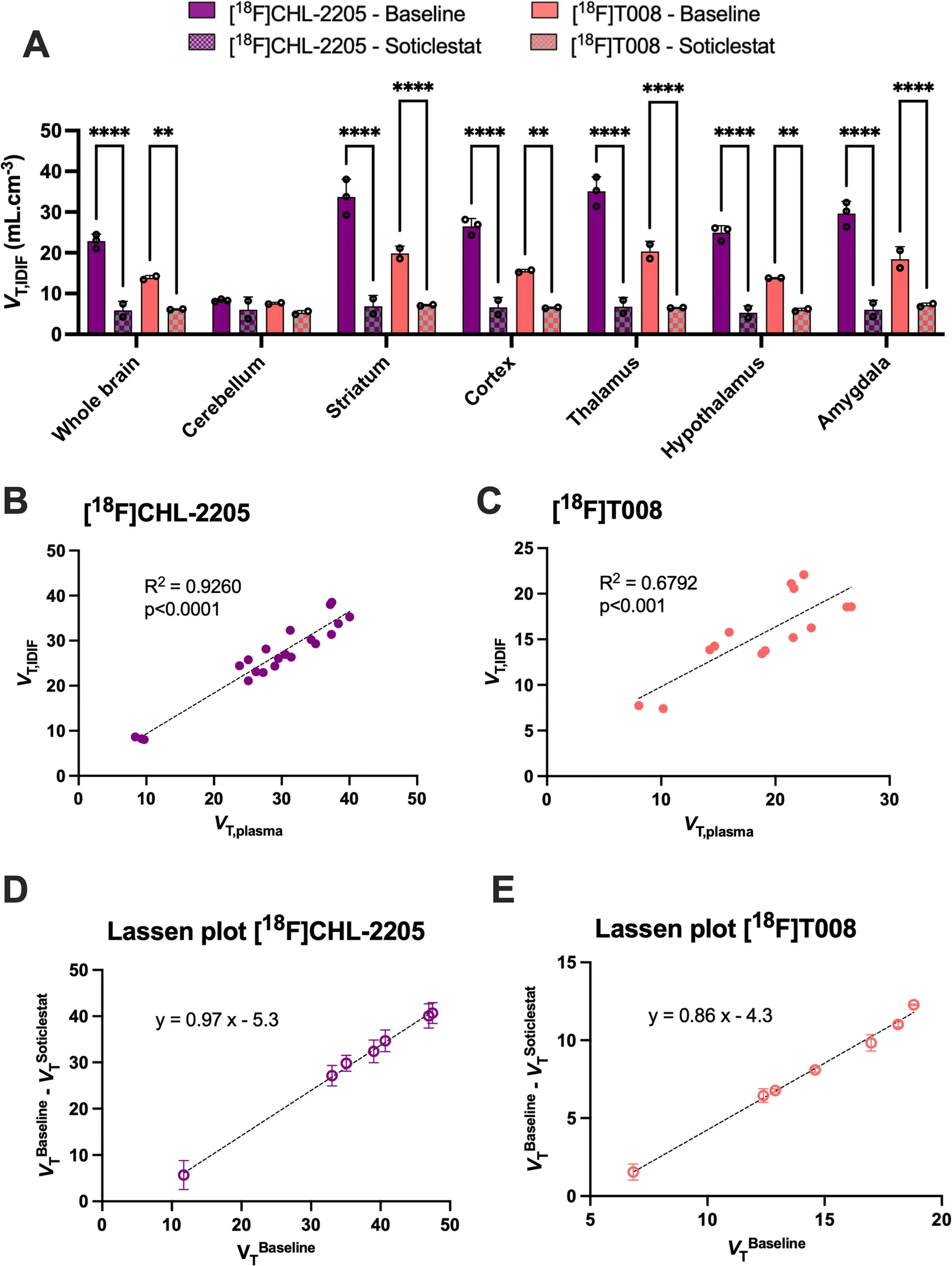
Validation of the image-derived input function (IDIF) and estimation of the specific binding of [^18^F]CHL-2205 versus [^18^F]T008 in rats. Baseline brain PET acquisitions were performed with [^18^F]CHL-2205 (*n* = 3) or [^18^F]T008 (*n* = 2) with concomitant arterial blood sampling for the determination of individual metabolite-corrected arterial input function to estimate the regional brain volume of distribution (*V*_T,plasma_). VT,IDIF was estimated in the same individuals using an image-derived input function (IDIF) corrected by a mean radiometabolite profile. Correlation between regional *V*_T,plasma_ and VT,IDIF was tested using the Pearson’s correlation test. In **C**, a Lassen plot analysis was performed to estimate the target engagement (slope of the graph) 30 minutes after injection of the CYP46A1 ligand soticlestat (0.9 mg/kg) and the non-specific binding (y-intercept) of [^18^F]CHL-2205 (*n* = 2) or [^18^F]T008 (*n* = 2)

### PET imaging in healthy nonhuman primates and rats

#### Healthy nonhuman primates

Two adult male cynomolgus macaques (*Macaca fascicularis*, aged 7 years, 9.8–10.8 kg) were used for PET studies. Each individual underwent a T1-weighted brain MR acquisition on an Achieva 1.5T scanner (Philips Healthcare, France). Brain PET data were acquired on the Biograph Horizon (Siemens, TN, USA) under standard anesthesia and monitoring procedures [9]. Macaques underwent a total of 6 PET scans (90 min), 3 with [^18^F]CHL-2205 and 3 with [^18^F]T008. Data acquisition started with the i.v bolus injection of [^18^F]CHL-2205 (186.0 ± 69.5 MBq, 0.4 ± 0.1 µg/kg) or [^18^F]T008 (217.6 ± 4.5 MBq, 0.2 ± 0.1 µg/kg). During PET acquisition, arterial blood sampling was performed by cannulating the femoral artery, located opposite the saphenous vein, which was used for radioligand injection. During the 1.5-hour scans, 20 blood samples (0.3 mL each) were collected: 13 during the first 15 min, followed by 7 samples at 10-minute intervals. Larger blood samples (2–3 mL) were collected at 5, 15, 30, 60, and 90 min (for the 1.5-hour scans) and at 120 min (for the 2-hour scans) for metabolite analysis.

**Fig. 5:**
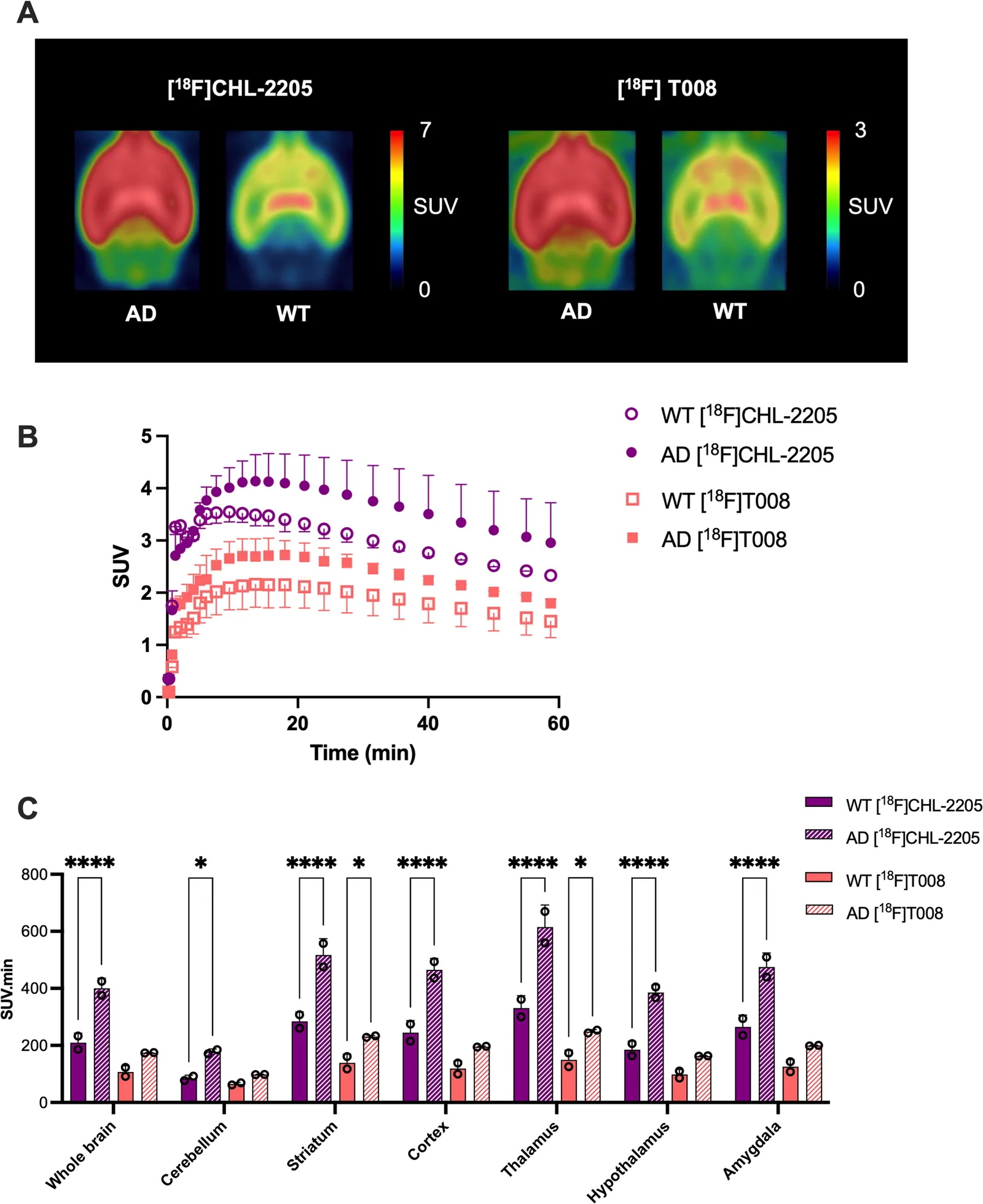
Comparison of [^18^F]CHL-2205 versus [^18^F]T008 in a rat model of Alzheimer’s disease (AD). Summed PET images obtained in TgF344-AD rats and in wild-type littermate (WT) are shown in **A**. Mean regional time-activity curves in the whole-brain are shown in **B**. Regional brain exposures (AUC.min, *n* = 2, mean ± S.D) obtained in each condition are displayed in **D**. Significant differences are shown as *p < 0.05 and ****p < 0.0001

#### Healthy rats

Brain PET data in rodents were acquired using two cross-calibrated Inveon microPET scanner (Siemens). Male Sprague-Dawley rats (age 6–8 weeks, 284 ± 30 g) were purchased from Janvier. PET acquisitions in rats were performed under standard anesthesia and monitoring procedures [10].

Baseline dynamic PET acquisitions started with the i.v bolus injection of [^18^F]CHL-2205 (46.8 ± 3.5 MBq, 2.5 ± 1.1 µg/kg, *n* = 3) or [^18^F]T008 (37.3 ± 5.4 MBq, 1.8 ± 0.3 µg/kg, *n* = 2) in the caudal vein. Arterial blood sampling was performed during PET acquisition by cannulating the femoral artery. Twenty blood samples (0.05 mL each) were collected: 12 during the first 10 min, followed by 8 samples at 5-minute intervals. Larger blood samples (0.10–0.15 mL) were collected at 5, 15, 30, and 60 min for metabolite analysis.

**Fig. 6:**
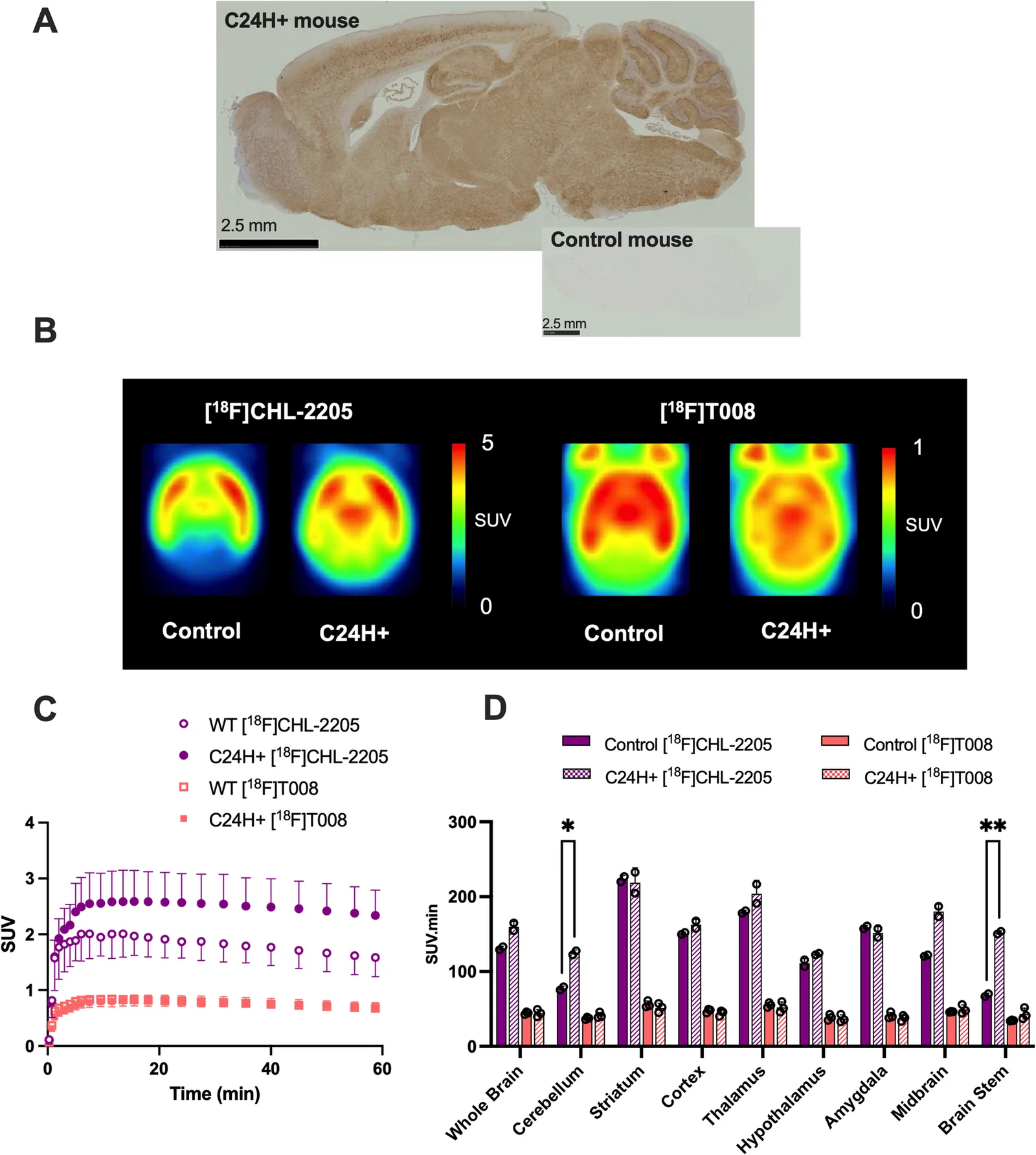
Comparison of [^18^F]CHL-2205 versus [^18^F]T008 in C24H-overexpressing mice. C24H-overexpressing (C24H+) mice were generated using a validated recombinant adeno-associated virus (AAV) vector. Hyaluronic acid staining on brain paraffin-embedded sections was performed as previously described [11]. Representative expression of the human CYP46A1 in transfected mice compared with control mice is shown in **A**. Representative summed PET images obtained in C24H+ mice and littermate controls are shown in B. Regional time-activity curves in the whole-brain are shown in **C**. Regional brain exposures (AUC.min, mean ± S.D) obtained in each condition are displayed in **D**. Significant difference is shown as *p < 0.05 and **p < 0.01

Four other Sprague-Dawley rats were used to compare the non-specific binding of [^18^F]CHL-2205 and [^18^F]T008. Pre-blocking studies were conducted using soticlestat at 0.9 mg/kg, administered 30 min before the radiotracer injection, which enables full target engagement in macaques [6]. Dynamic PET acquisition started with the i.v bolus injection of [^18^F]CHL-2205 (35.0 ± 1.4 MBq, 2.6 ± 0.2 µg/kg, *n* = 2) or [^18^F]T008 (41.5 ± 9.2 MBq, 5.5 ± 2.0 µg/kg, *n* = 2). No arterial blood sampling was performed during pre-blocking scans.

#### Arterial input function and radiometabolite analysis

Blood samples were collected during PET acquisition in healthy macaques and rats. Plasma was separated from whole blood by centrifugation (5 min, 2054 g, 4 °C). Plasma and whole-blood samples were counted using a PET cross-calibrated gamma well counter (WIZARD2, PerkinElmer, France). All data were corrected for radioactive decay from the time of injection.

For radiometabolite analysis, plasma was deproteinized with acetonitrile. The supernatant was injected into a high-performance liquid chromatography (HPLC) system equipped with an Atlantis^®^ T3 5 μm 4.6 × 250 mm column (Waters, France) and an Atlantis^®^ T3 5 μm 3.9 × 5 mm pre-column (Waters), with an LB-513 radioactivity flow detector (Berthold, France). The parent fraction was calculated as a percentage of total radioactivity (parent and metabolites).

### Brain PET imaging in murine models with C24H overexpression

#### Rat model of Alzheimer’s disease

The TgF344-AD rat line, which expresses two mutant human genes: the “Swedish” APP (APPsw) and the Δ exon 9 PS1 (PS1ΔE9), is a widely used model of AD [12]. Two male TgF344-AD rats and 2 male wild-type (WT) littermate breeding pairs (age 59–60 weeks, 269 ± 28 g) were purchased from the group of Prof. Terrence Town at the University of Southern California who developed this strain [12]. These animals were set up as breeding pairs, housed in the Biological Services Unit at the University of Manchester. Breeding pairs from this colony were sent to the animal facility of our institute, where TgF344-AD and WT littermates were bred. Genotyping was outsourced to the Genotyping and Biochemistry Center to confirm the mutated genotype in each individual (CGB, Paris, France).

Sixty-minute brain microPET acquisitions were performed for each animal, with *n* = 2 receiving [^18^F]CHL-2205 and *n* = 2 receiving [^18^F]T008 for each condition. Data acquisition started with the i.v bolus injection of [^18^F]CHL-2205 (33.3 ± 5.0 MBq, 1.9 ± 0.6 µg/kg) or [^18^F]T008 (33.8 ± 7.1 MBq, 2.7 ± 1.3 µg/kg).

#### C24H-overexpressing mice

C24H-overexpressing mice were generated using a recombinant adeno-associated virus (AAV) vector administered in male C57BL/6JRj mice (age 10–12 weeks, 21.7 ± 1.2 g, Janvier, France). The AAVPHP.eB-CYP46A1-HA vector was produced by Atlantic Gene Therapies (INSERM U1089, Nantes, France) following established protocols [13]. This vector contains the human *CYP46A1* gene with an HA (hyaluronic acid) tag, driven by a synthetic CAG promoter and flanked by AAV2 ITR sequences [14]. Mice received 5.10¹¹ vg of the vector (100 µL diluted in 0.9% NaCl) via retro-orbital injection under isoflurane anesthesia. We have shown that this model enables expression of human C24H in the brain, in addition to endogenous murine C24H expression [11, 13].

PET imaging was performed 15 days after transfection. Sixty-minute brain microPET acquisitions were performed with [^18^F]T008 in three C24H-overexpressing and three control (wild-type) mice. Two of these C24H-overexpressing and 2 of the control mice underwent another PET scan with [^18^F]CHL-2205. Data acquisition started with the i.v bolus injection of either [^18^F]CHL-2205 (10.5 ± 1.0 MBq, 10.6 ± 2.8 µg/kg) or [^18^F]T008 (10.9 ± 1.6 MBq, 12.0 ± 4.8 µg/kg).

### PET data reconstruction and analysis

Dynamic PET images were reconstructed using standard OSEM-3D algorithms, corrected for radioactive decay, scatter, attenuation, and detector inhomogeneity. PET image analysis was performed using PMOD software version 4.404 (PMOD Technologies Ltd., Zurich, Switzerland).

For macaques, brain PET data were coregistered to individual brain T1 MR images. Then, a cynomolgus macaque atlas [15] was normalized to individual MR images and coregistered to PET images to extract time-activity curves (TACs) for selected brain regions. For rats, co-registration was performed using the W. Schiffer template, and for mice, the Ma-Benveniste-Mirrione template was used. TACs were expressed as standardized uptake values (SUV) *versus* time (min). Brain exposure was estimated by calculating the area under the TACs (SUV.min) for the total duration of the PET acquisition.

Pharmacokinetic modeling of brain PET data obtained in macaques and rats was performed using Logan graphical analysis with arterial input function to estimate the total volume of distribution (*V*_T, plasma_) in each brain region. In healthy rats, an image-derived input function (IDIF) was tested to estimate *V*_T,IDIF_. To this end, a volume-of-interest was drawn on the left-heart ventricle, and corresponding TACs were corrected by the mean plasma-to-whole blood ratio and the mean metabolism profile of [^18^F]CHL-2205 or [^18^F]T008.

*V*_T_,_IDIF_ values estimated in healthy rats in the baseline (*n* = 3 for [^18^F]CHL-2205 and *n* = 2 for [^18^F]T008) and the pre-blocking conditions (*n* = 2 for [^18^F]CHL-2205 and *n* = 2 for [^18^F]T008) were used to estimate the specific binding in brain regions. The binding potential (BP_ND_) BP_ND_ = *V*_T_ / *V*_ND_ – 1, where *V*_ND_ is the non-displaceable volume of distribution [16]. In the absence of an ideal reference region devoid of the CH24H enzyme, *V*_ND_ was estimated using a Lassen plot using the data of the pharmacological blocking study achieved following a 0.9 mg/kg dose of soticlestat [4, 6]. The equation used was:

$$V_{\mathrm T,\mathrm{Baseline}}-V_{\mathrm T,\mathrm{Soticlestat}}=O_{\mathrm{cc}}\times\left(V_{\mathrm T,\mathrm{Baseline}}-V_{\mathrm{ND}}\right)$$

where *V*_T, Baseline_ and *V*_T, Soticlestat_ (IDIF-derived) are the total volumes of distribution at baseline and after a saturating dose of soticlestat, respectively, and O_CC_ represents the global occupancy. The x-intercept of the plot was used to estimate the non-displaceable distribution (*V*_ND_). The occupancy (%) was estimated by the slope of the Lassen plot correlation [17].

## Results

### [^18^F]CHL-2205 vs. [^18^F]T008 in healthy macaques

#### Brain kinetics

Figure 2A displays representative brain PET images obtained in macaques after injection of [^18^F]CHL-2205 or [^18^F]T008. For both [^18^F]CHL-2205 and [^18^F]T008, the highest uptake was observed in the putamen, and the lowest was in the cerebellum. In the putamen, both tracers progressively entered the brain, reaching maximum uptake at ~ 50 min post-injection for [^18^F]T008 *versus* ~ 80 min for [^18^F]CHL-2205, followed by a slow washout from the brain. In the cerebellum, both tracers entered the brain rapidly, achieving peak uptake 4 min after injection, followed by a relatively slow washout (Fig. 2B).

#### Blood kinetics

[^18^F]CHL-2205 and [^18^F]T008 exhibited similar metabolism profile in arterial blood: the parent (unmetabolized) fraction was 62.2 ± 9.0% for [^18^F]CHL-2205 *versus* 51.0 ± 4.0% for [^18^F]T008 at 15 min post-injection, 38.6 ± 5.6% for [^18^F]CHL-2205 *versus* 37.3 ± 9.0% for [^18^F]T008 at 60 min, and 28.5 ± 8.0% for [^18^F]CHL-2205 *versus* 33.5 ± 2.6% for [^18^F]T008 at 90 min post-injection (Fig. 2C). The parent fraction was fitted to a mono-exponential decay function. Mean metabolite-corrected arterial input functions obtained with each radiotracer are shown in Fig. 2D. The plasma-to-whole-blood ratio was 0.84 ± 0.02 for [^18^F]CHL-2205 *versus* 0.90 ± 0.03 for [^18^F]T008 and was constant over the 90-minute acquisition period (Supp. Fig S1).

#### Kinetic modeling

Figure 2E displays the *V*_T_ values derived from kinetic modeling of blood and brain data for both tracers in predefined brain regions. On average, regional brain *V*_T_ values for [^18^F]CHL-2205 were ~ 1.6-fold higher [1.2- to 1.7-fold across brain regions] than those of [^18^F]T008 obtained in the same individuals, and significant in the caudate and putamen (*p* < 0.001), the amygdala (*p* < 0.01) and the frontal cortex (*p* < 0.05).

### [^18^F]CHL-2205 vs. [^18^F]T008 in healthy rats

#### Brain kinetics

Figure 3A displays representative brain PET images obtained in healthy rats after injection of [^18^F]CHL-2205 or [^18^F]T008. Mean TACs obtained in the thalamus and cerebellum are shown in Fig. 3B. For both radiotracers, the highest uptake was observed in the thalamus, and the lowest uptake was seen in the cerebellum. In the thalamus, both tracers progressively entered the brain, reaching maximum uptake at 18–21 min for [^18^F]CHL-2205 and 15–21 min post-injection for [^18^F]T008, followed by a slow washout from the brain. In the cerebellum, both tracers entered the brain rapidly, achieving peak uptake 5 min after injection, followed by a relatively slow washout.

#### Blood kinetics

In rats, [^18^F]CHL-2205 and [^18^F]T008 exhibited similar and rapid metabolism in arterial blood, with an intact parent fraction of 24.8 ± 3.3% for [^18^F]CHL-2205 and 23.8 ± 1.8% for [^18^F]T008 at 15 min post-injection (Fig. 3C). The parent fraction was 7.6 ± 0.6% for ^[18F^]CHL-2205 and 9.8 ± 1.5% for [^18^F]T008 at 60 min post-injection. The parent fraction was fitted to a two-exponential decay function based on blood data acquired up to 1 h post-injection (Fig. 3C). Mean arterial input functions obtained with each radiotracer (*n* = 2 for [^18^F]CHL-2205 and *n* = 3 for [^18^F]T008) are shown in Fig. 3D. The plasma-to-whole-blood ratio was 0.82 ± 0.07 for [^18^F]CHL-2205 and 0.98 ± 0.03 for [^18^F]T008 over the 60-min acquisition period (Suppl. Fig. S1).

#### Kinetic modeling

Figure 3E displays the *V*_T_ values estimated from kinetic modeling of [^18^F]CHL-2205 and 0.98 ± 0.03 for [^18^F]T008 in predefined brain regions of healthy rats. On average, *V*_T_ values for [^18^F]CHL-2205 were ~ 1.5-fold higher than those obtained with [^18^F]T008 across all regions, except for the cerebellum, where no significant difference was observed.

*V*_T, IDIF_ values estimated with the IDIF in the baseline and pre-blocking conditions are shown in Fig. 4A. *V*_T, IDIF_ values obtained for [^18^F]CHL-2205 and [^18^F]T008 with the IDIF in the baseline condition were strongly correlated with *V*_T_ values obtained with the arterial input function in the same animals (Fig. 4B and C), thus validating this minimally-invasive quantification method.

*V*_T, IDIF_ values estimated with the IDIF in the baseline and pre-blocking were used to estimate *V*_ND_ using the Lassen plot analysis (Fig. 4D and E). Using the same dose of soticlestat, the estimated global occupancy was 97% for [^18^F]CHL-2205 and 86% for [^18^F]T008 (*n* = 2 per radiotracer), suggesting almost total occupancy of the available C24H. *V*_ND_ values estimated from the x-intercept of the Lassen plot were 5.3 mL.cm^− 3^ for [^18^F]CHL-2205 and 4.3 mL.cm^− 3^ for [^18^F]T008. Estimated regional BP_ND_s for [^18^F]CHL-2205 and [^18^F]T008 are reported in Table 1. These data confirm a trend toward higher BP_ND_ values for [^18^F]CHL-2205 compared to [^18^F]T008 in most brain regions except the cerebellum (Table 1).

**Table 1:** Estimation of the specific binding of [¹⁸F]CHL-2205 and [¹⁸F]T008 from the Lassen plot graphical approach in control rats

Regions	BP_ND_ values for [^18^F]CHL-2205 (*n* = 3)	BP_ND_ values for [^18^F]T008 (*n* = 2)	*p*-value
Whole brain	3.7 ± 0.2	2.9 ± 0.7	0.3704
Cerebellum	0.7 ± 0.1	1.1 ± 0.3	0.9551
Striatum	6.0 ± 0.3	4.6 ± 0.9	0.0267 *
Cortex	4.5 ± 0.3	3.4 ± 0.9	0.0976
Hippocampus	6.2 ± 0.3	4.7 ± 0.6	0.0098 **
Thalamus	4.1 ± 0.4	2.9 ± 0.8	0.0467 *
Amygdala	5.1 ± 0.3	4.2 ± 0.2	0.2787

The binding potential (BP_ND_) is reported as a mean±S.D (*n* = 2-3) for both radiotracers. Significant differences are shown as *p < 0.05 and **p < 0.01

### [^18^F]CHL-2205 vs. [^18^F]T008 in a rat model of Alzheimer’s disease

Figure 5A shows representative brain PET images obtained using [^18^F]CHL-2205 and [^18^F]T008 in a rat model of AD and in corresponding wild-type (WT) littermates. Using both radiotracers, a significant increase in brain uptake was observed in the rat model of AD compared to WT rats. In the whole-brain, [^18^F]CHL-2205 showed an average 1.9-fold increase in brain uptake in AD rats compared to WT rats, whereas [^18^F]T008 exhibited an average 1.6-fold increase under the same conditions (Fig. 5B).

### [^18^F]CHL-2205 vs. [^18^F]T008 in C24H-overexpressing mice

Ex vivo staining experiments confirmed the broad CYP46A1-HA overexpression in the brain of transfected mice (C24H+) compared to non-injected mice (Fig. 6A). Consequently, CYP46A1-HA expression in transfected mice comes in addition to the baseline murine Cyp46a1 expression, thus providing a mouse model of increased target availability.

Figure 6B displays representative microPET images obtained from C24H-overexpressing mice compared to control (non-transfected) mice. Using [^18^F]T008, no differences in radioactivity uptake were observed between control and mice C24H-overexpressing, except in the brainstem, which showed a non-significant 1.25-fold increase (*p* > 0.05, *n* = 3 per condition, Fig. 6C and D). Two of these C24H-overexpressing mice (same individuals) underwent subsequent [^18^F]CHL-2205 PET scans that showed increased radioactivity uptake in the thalamus (1.13-fold, *p* > 0.05), cerebellum (1.61-fold, *p* < 0.05), brainstem (2.22-fold, *p* < 0.01), central gray (1.53-fold, *p* > 0.05), and midbrain (1.48-fold, *p* > 0.05) compared to control mice (*n* = 2; Fig. 6C and D).

## Discussion

In the present study, brain PET data were acquired using two different C24H-targeting radiopharmaceuticals in multiple parallel conditions to compare their brain kinetics and assess their relative sensitivity in detecting increases in C24H expression.

The first C24H-targeting PET radioligands were introduced in 2022 as much-needed tools to study brain cholesterol homeostasis in vivo [4, 7]. The aryl-piperidine derivatives [^18^F]CHL-2205 and [^18^F]T008 share a similar chemical structure, differing only in the tertiary amide substituents (Fig. 1). These radiotracers have undergone extensive preclinical validation across multiple laboratories using dedicated animal models. Both radioligands exhibit highly favorable PET kinetic profiles for studying cholesterol homeostasis in the living brain. Moreover, [^18^F]CHL-2205 and [^18^F]T008 have been successfully transferred to humans and are now available for studying the importance of brain cholesterol homeostasis in various neurological conditions, including AD [3]. However, these tracers need to be compared under parallel conditions to guide the choice between them for future translational research projects.

Despite similar chemical structures, the position of fluorine-18 radiolabeling differs, which may affect the proportion of radiometabolites in plasma [18]. However, our results in Cynomolgus macaques and rats show that [^18^F]CHL-2205 and [^18^F]T008 have very similar metabolism profiles and plasma kinetics. This contrasts with the higher brain uptake of [^18^F]CHL-2205, which results in higher regional *V*_T_ values compared with [^18^F]T008. The relative distribution of PET radiotracers across brain regions, as well as higher regional *V*_T_ values in Cynomolgus macaques, are consistent with previous data from different labs on [^18^F]CHL-2205 and [^18^F]T008 in Rhesus macaques [4, 6].

[^18^F]CHL-2205 and [^18^F]T008 are aryl-piperidine derivatives with different chemical structures (Fig. 1). Different in vitro affinities for C24H have been reported (K_d_ = ~ 0.3 nM for [^18^F]CHL-2205 versus K_d_ = 14 nM for [^18^F]T008), although these data were obtained in different conditions [4, 7]. Reported BP_ND_ values, which describe the target-specific binding, were acquired in different labs and were ~ 22 for [^18^F]CHL-2205 [4] vs. ~ 15 for [^18^F]T008 [6] in the putamen. To address these potential differences, kinetic modeling was performed in rats under the same conditions and in the same laboratory, in the absence and presence of a saturating dose of soticlestat. Estimated BP_ND_ values were higher for [^18^F]CHL-2205 compared to [^18^F]T008, indicating higher specific binding in brain regions.

C24H-deficient mice and saturation using soticlestat were useful to validate the specificity of [^18^F]CHL-2205 and [^18^F]T008 for C24H in vivo, showing a significant decrease in the PET signal when the target is absent or pharmacologically engaged [4, 6]. However, a large body of research suggests that C24H expression and function are enhanced in AD patients as an attempt to eliminate the excess brain cholesterol [3]. Consistently, the brain PET signal associated with [^18^F]CHL-2205 was shown to be higher in 3xTg-AD mice relative to control mice [4]. In the present study, the relative sensitivities of [^18^F]CHL-2205 and [^18^F]T008 to detect increases in C24H expression were tested in two rodent models. First, in TgF344-AD rats relative to their WT littermates, the increase in brain uptake was higher with [^18^F]CHL-2205 than with [^18^F]T008, suggesting greater sensitivity to detect increases in C24H expression. This was confirmed in human C24H-overexpressing mice in which [^18^F]T008 failed to detect differences in human C24H expression in the brain, which was possible in certain brain regions using [^18^F]CHL-2205.

This study has some limitations. Full kinetic modeling was not performed in TgF344-AD and C24H-overexpressing mice because the metabolite-corrected IDIF method cannot be straightforwardly applied in different rat strains or in mice without validation. Moreover, the number of animals in each condition is relatively small (*n* = 2–3). As a future perspective for this work, it would be valuable to assess C24H expression levels in the same individuals after PET imaging to enable direct in vivo/ex vivo correlation and further validate the findings.

[^18^F]CHL-2205 and [^18^F]T008 have undergone extensive preclinical-to-clinical validation and are now being used in several clinical trials worldwide. Research in medicinal chemistry continues to propose alternative radioligands to investigate C24H in the living brain [19, 20]. However, these ligands still need to be validated for clinical use, and their added value remains to be demonstrated.

## Conclusion

In this preclinical study, the relative performance of [^18^F]CHL-2205 and [^18^F]T008 was compared across complementary preclinical models, demonstrating the superiority of [^18^F]CHL-2205 for detecting subtle increases in C24H expression in the living brain, likely due to its higher specific binding in the brain. These preclinical data, obtained in rodents and non-human primates, may help guide the selection of a radiotracer when designing a preclinical or clinical study to investigate brain cholesterol metabolism using C24H PET imaging.

## Electronic Supplementary Material

Below is the link to the electronic supplementary material.


Supplementary Material 1 (PDF 188 KB)


## Data Availability

The datasets generated during and/or analyzed during the current study are available from the corresponding author on reasonable request.
