# Supplementary material for: Head-to-head comparison of [18F]CHL2205 versus [18F]T008 as PET radioligands to study brain cholesterol homeostasis in rodent models and nonhuman primates
Source: Eur J Nucl Med Mol Imaging. 2026 May 30;53(11):6154–66. doi: 10.1007/s00259-026-07937-9 (PMC13526071; doi:10.1007/s00259-026-07937-9)
Supplement: Supplementary file 1 [file 259_2026_7937_MOESM1_ESM.pdf]

Supplementary Information

Head-to-head comparison of [^{18}F]CHL2205 *versus* [^{18}F]T008 as PET radioligands to study brain cholesterol homeostasis in rodent models and nonhuman primates

Sébastien GOUTAL, Fabien CAILLE, Françoise PIGUET, Maud GOISLARD, Sophie AMARGIER-BARRIAL, Sarah LETERRIER, Solène MARIE, Michel BOTTLAENDER, Nicolas TOURNIER

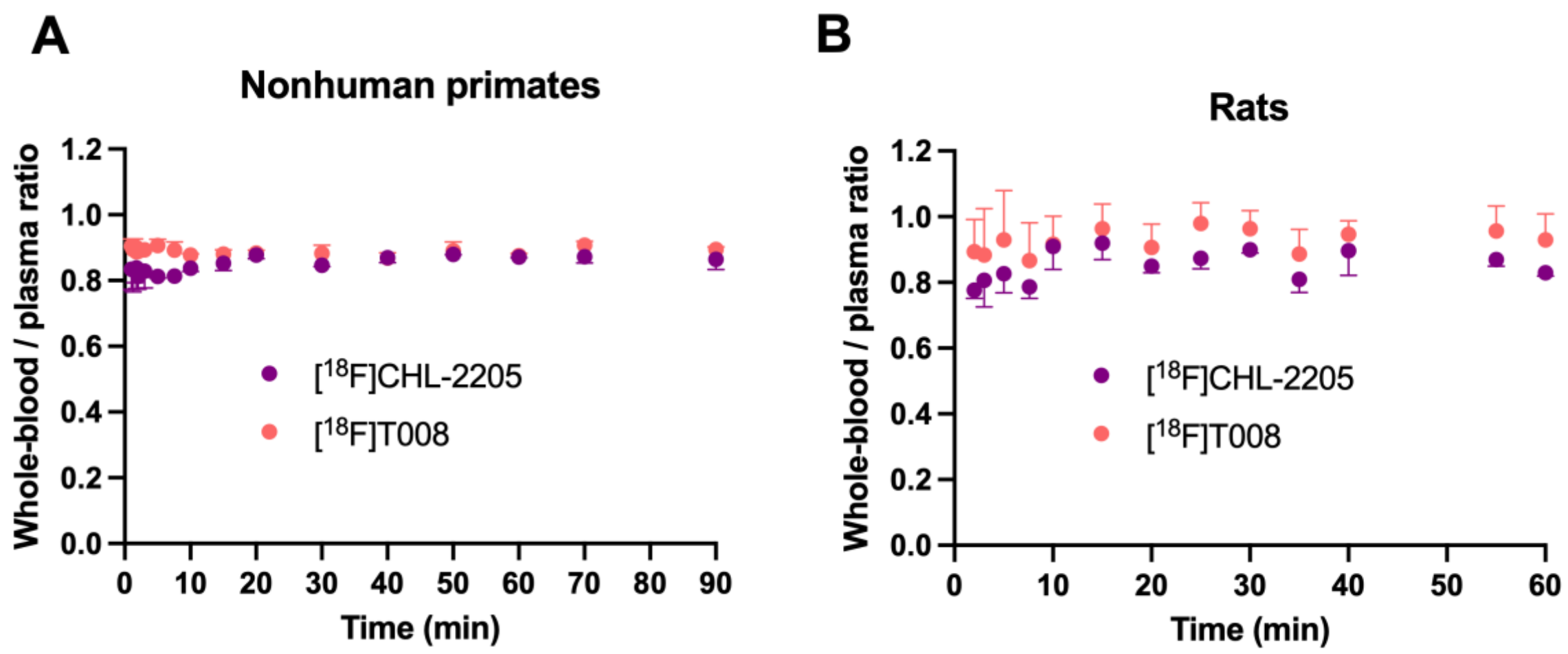

Supp. Fig. S1. Time-course of the whole-blood to plasma ratio of [^{18}F]CHL2205 and [^{18}F]T008 observed in nonhuman primates (A) and in rats (B). Blood sampling was performed during PET acquisition. Data are reported as mean ± 1 S.D with n=3 experiments for each radiotracer in nonhuman primates, n=3 for [^{18}F]CHL2205 in rats, and n=2 for [^{18}F]T008 in rats.
